# Electro-Therapeutics

**Published:** 1903-09

**Authors:** J. Taylor


### ELECTRO-THERAPEUTICS.

The **high-frequency electrical currents** and the X-rays up to the present have generally been classed together as similar methods of treatment; thus we find in the *Encyclopædia Medica*

the chapter on high-frequency currents under the heading of X-rays. This is not as it should be, and the two methods should be kept distinct. Lewis Jones[1] points out that they have one feature in common, viz. the Cathode rays, and says that perhaps in the future we may prefer to talk of "treatment by Cathode rays"; but as the results of the high-frequency currents—at any rate in the treatment of general diseases—depend upon the electrical phenomena and upon the physiological effects produced, the method should be looked upon as a different mode of treatment from that of the X-rays, whose action is mainly local, and dependent on the reaction produced on the diseased part.

Unfortunately, the treatment by the high-frequency currents has become to a certain extent obnoxious to the profession, as it has been exploited by the daily press, seized upon by tradesmen running establishments for the cure of every ill that human flesh is heir to by electrical means, and has even formed part of a music hall performance in which the performer professed to cure paralysis. Small wonder, therefore, that the method should be looked at askance by some members of the profession. Still, the method is of distinct value in certain cases, and it should be investigated by properly qualified men, especially as to the class of disease in which it is useful, and its proper mode of application, dosage, etc. Above all, the remedy should be kept by practitioners in their own hands. It is not right that patients should be sent for treatment to some enterprising tradesman who has set up an X-ray installation for the purpose of taking radiograms, and has added a high-frequency apparatus to his stock-in-trade, for the remedy is one that wants watching by one who has a practical knowledge of the disease for which the treatment is applied.

**Diabetes.**—The marked physiological effects produced by the high-frequency currents necessarily led to their use in general diseases, such as gout, rheumatism, diabetes, etc., and the results attained up to the present, as reported by different observers, differ very much, some speaking of the treatment as most efficacious, and others that it is of little use. Thus four cases of diabetes have been recorded by Vietta and two by D'Arsonval,[2] in which the sugar either disappeared or was much reduced. Chisholm Williams[3] gives notes of a case where the patient was passing 16 pints of urine in the 24 hours, and 32·3 grains of sugar to the ounce when the treatment was commenced, but at the end of a month's treatment the urine had decreased to 7 pints, and only 3 grains of sugar per ounce. Against these favourable reports Lewis Jones,[4] quoting Dénoyés, says that the patients still remain glycosuric, and that reduction of the sugar can be obtained equally well by other electrical means, such as the electrical bath with sinusoidal current.

[1] *Practitioner*, 1903, lxx. 361. [2] *Ency. Med.*, xiii. 529.
[3] *The High-frequency Currents in the Treatment of Disease*, 1903, 159. [4] *Loc. cit.*

Apparently, therefore, reduction in the quantity of the sugar excreted in cases of diabetes is acknowledged alike by the supporters and the detractors of the treatment, and as reduction of the sugar excreted is the object aimed at in all other treatments of diabetes, surely this method is worthy of extensive trial. It certainly has the advantage over electric baths in being applied much more easily, and at infinitely less trouble to the patient.

**Tubercular Disease.**—Dénoyés' experimental work on guinea pigs, the details of whose work are given at length in his work on "Les Courants de haute fréquence," and whose results are quoted in most English articles on the subject, shows that the high-frequency currents have a retarding influence on the growth of the tubercle bacillus; and Rivière[1] states that "the microbe cannot resist the repeated application of these currents, its reproductive power and the virulence of its toxins being attenuated." Again, Grünbaum,[2] discussing the theory of immunity, points out that the modification from toxin to toxoid may be brought about by the high-frequency currents, and that toxoids are capable of producing immunity. In the face of such statements as these, is it too much to expect that the high-frequency currents will have a beneficial effect in the treatment of phthisis? and will they not prove a valuable adjunct to the open-air treatment of the disease? According to Chisholm Williams[3] they have such an effect, and in his lately published work he gives further information of the forty-three cases he originally recorded. Of these three have died, the three deaths being due to pneumonia, tuberculous kidney, and lardaceous disease; of the rest, thirty-two have had no treatment of any kind for eighteen months, eight cases have had on an average two months' treatment since that time, and none of them have required treatment during the past year. The majority of the patients, who were workers, are performing their usual duties. As he suggests, "when many hundreds of phthisical patients have been subjected to this method we shall be in a position to judge of its merits."

In the treatment of **malignant disease** some cases have been reported in which improvement has been obtained by the use of the currents, notably four cases reported by Dr. Allan,[4] viz. one of sarcoma of the antrum, two of recurrent scirrhus of the breast, and one of advanced epithelioma of the os uteri. All of these cases were remarkably relieved, and the disease apparently arrested. A further report of these cases is given in an appendix in Chisholm Williams's work on the high-frequency currents, stating that three of the patients were quite well two years after the treatment, whilst the fourth (epithelioma of the uterus) had died of pneumonia. Other observers have not been so fortunate in their results, the general opinion being that though patients

[1] *Brit. M. J.*, 1902, i. 93. [2] *Ibid.*, 1903, i. 654. [3] *Op. cit.*
[4] *West Lond. M. J.*, 1902, vii. 173.

are improved in their general health for the time, yet in spite of the temporary improvement the disease goes on in the usual manner to a fatal conclusion. I myself have seen one extraordinary case of great improvement in the symptoms under the use of the currents in a case of pelvic carcinoma. The patient had a mass of malignant growth in Douglas's pouch involving the rectum and vagina, which could be felt above the pubes. She had great distress in defæcation, the motions being blood-stained and only passed with the greatest difficulty, and her pain and distress were only kept under control by large doses of morphia frequently repeated. After four months' treatment with the high-frequency currents, administered by an electrode in the rectum, she lost all pain, entirely gave up the use of morphia, passed natural motions, and was apparently in perfect health, and able to live her usual life. The improvement commenced after the first few weeks of the treatment. The tumour, however, remained much as before, and probably the improvement is only for a time. However, if the chance of cure is only a remote one, still in this otherwise certainly fatal disease the remedy is worth while trying. The report by the Middlesex Hospital authorities on the subject of treatment of cancer by the high-frequency currents, X-rays, etc., is anxiously awaited by the profession.

In **chronic rheumatism** the remedy is most useful, especially in rheumatic arthritis of single joints, the pain being greatly relieved and the movements being improved. It is well known that the continuous electric current has been very useful in this class of cases, but the high-frequency currents seem to be much more effectual and the effect more lasting. In rheumatoid arthritis also it seems to improve mobility of the joints and to lessen pain.

In **chronic neuralgias,** such as the persistent neuralgia which follows an attack of herpes zoster, the remedy seems of distinct value.

It is, however, in the more **local diseases** that the high-frequency currents have their greatest use. All rectal troubles seem to be greatly benefited by the treatment, more especially fissure of the anus, and pruritus ani. The former disease is speedily cured, but probably the passage of the electrode stretching the sphincter has a good deal to do with the cure in these cases. But the relief afforded by the currents in cases of long-standing pruritus is sometimes remarkable. Thus I have known one patient say after four applications that he was more comfortable than he had been for twenty years.

In the treatment of **hæmorrhoids** the remedy is useful, but is not a specific as has been supposed. In recent cases a cure is rapidly brought about, but in chronic cases it takes a long course of treatment before any improvement is noticed. Hinsley Walker[1] seems to have been very successful in the treatment of

[1] *Brit. M. J.*, 1902, i. 93.

his cases; but Chisholm Williams[1] says that thirty applications are required before improvement is noted—his remarks "only apply to patients who from age or other cause refuse operation."

The treatment is well spoken of as a remedy in **skin diseases.** Regnier[2] says that cases of eczema readily respond to the treatment, also some cases of psoriasis. Chisholm Williams[3] notes a curious fact, that warts can be made to disappear with sparking the growth with a small-pointed electrode.

In that distressing complaint—at least to other people if not to the patient—**ozæna**, I have seen one case in which the offensive smell was entirely cured after a fortnight's application of the effleuve from a nasal electrode passed up the nostrils. The patient was a young woman of twenty, and the disease had been present since early childhood. J. TAYLOR.

### Reviews of Books.

[1] *Op. cit.*, 196. [2] *Progrès Méd.*, 1902, 3 s. xv. 316. [3] *Op. cit.*, 159.

